# Effects of recurrent ketamine exposure on brain histopathology in juvenile rats

**DOI:** 10.55730/1300-0144.5554

**Published:** 2022-09-12

**Authors:** Ayşe Hande ARPACI, Süheyla Esra ÖZKOÇER, Emel GÜNEŞ, Çiğdem ELMAS, Berrin IŞIK

**Affiliations:** 1 Department of Oral and Maxillofacial Surgery, Faculty of Dentistry, Ankara University, Ankara, Turkey; 2 Department of Histology and Embryology, Faculty of Medicine, Gazi University, Ankara, Turkey; 3 Department of Physiology, Faculty of Medicine, Ankara University, Ankara, Turkey; 4 Department of Anesthesiology and Reanimation, Faculty of Medicine, Gazi University, Ankara, Turkey

**Keywords:** Brain injury, apoptosis, cleaved caspase-3, ketamine, juvenile rat

## Abstract

**Background/aim:**

Ketamine (KET) is a commonly used anesthetic agent. However, several previous studies reported that KET leads to neuronal damage in neurodevelopmental stages and has neuroprotective effects. The present experimental study aimed to determine the undesirable histopathological effects of KET in the cerebral cortex, striatum, and hippocampus after recurrent KET administration in juvenile rats.

**Materials and methods:**

After ethical approval was obtained, 32 juvenile male Wistar Albino rats were randomized into four groups: 1 mg/kg serum saline intraperitoneally (i.p.), 5 mg/kg KET i.p., 20 mg/kg KET i.p., and 50 mg/kg KET i.p. KET was administered for three consecutive days at three-h intervals in three doses. Ten days after the last KET dose, the rats were sacrificed. Cerebral hemispheres were fixed. Hematoxylin and eosin stain was used for morphometric analysis. Hippocampi were evaluated by immunohistochemistry with anticleaved caspase-3 antibodies. Statistical analysis was conducted with SPSS 21 software using the ANOVA test and Bonferroni post hoc analysis method.

**Results:**

The experimental study findings revealed no difference between the groups’ cell counts or sizes in cortical morphometry. No degenerative changes were observed in pyramidal and granular cells in the striatum. Mild gliosis was observed in the 20 mg/kg and 50 mg/kg KET administration groups. Immuno-histo-chemical analysis was conducted to determine apoptosis in the CA1 region of the hippocampus and revealed that caspase-3 positivity increased with the KET dose. However, there was no statistical difference between the groups. While it was lower than the control group in the 5 mg/kg KET group, it was similar to the control group in the 20 mg/kg KET group and higher in the 50 mg/kg KET group (p > 0.05).

**Conclusion:**

Repetitive KET exposure did not significantly affect juvenile cerebral morphology and apoptosis in hippocampal cells.

## 1. Introduction

Ketamine (KET) was synthesized as an anesthetic agent by Calvin Stevens in 1962 and was initially used by Corssen & Domino in 1965. It has been widely employed in clinical practice since the 1970s [1]. It is the only agent with hypnotic (sleep-inducing), analgesic (pain-relieving), and amnesic (short-term memory loss) effects; thus, it is a “unique drug” [1]. It leads to dissociative anesthesia by noncompetitive blocking of N-methyl-D-aspartate acid and glutamate receptors [2]. It is widely used, especially in children, due to its rapid onset, short functional duration, hemodynamic safety, upper respiratory tract reflex protection, lack of respiratory depression, and strong analgesic properties. Currently, it is even used as an opioid-induced respiratory depression antagonist in treating chronic pain and treatment-resistant depression [2–6].

However, several studies demonstrated that KET could induce damage, especially in developing brain tissue, which could lead to behavioral changes later in life [7–11]. In the neonatal period, KET was reported to increase neuronal necrosis in the brain based on the dose and exposure, and it was reported to cause neuronal defects in the long term [12,13]. Experimental studies in rodents and nonhuman primates also report increased apoptosis in developing brain tissues and triggered necrosis [14–22]. Other studies demonstrated that KET induced apoptosis in the sensorimotor cortex and cerebellum in a dose- and exposure-dependent manner [23].

Caspases, an important gene family, are effective in maintaining homeostasis through the regulation of necrosis and inflammation in apoptosis mechanisms [24–27]. Caspases 3, 6, 7, 8, and 9 are associated with apoptosis, and caspases 1, 4, 5, and 12 are associated with inflammation. Cleaved caspase-3 is a reliable marker for cells that are dying or have already died due to apoptosis [28]. Clinical studies reported that the caspase-3 level affected the determination of the prognosis and local recurrence in cancer cases [29–31].

On the other hand, although it is known that exposure to an anesthetic KET dose increases inflammation, autophagy, apoptosis, and reactive oxygen species levels in neurodevelopmental stages, the neuroprotective properties of KET at subanesthetic doses have also been demonstrated [32].

It is unclear for how long and to what extent recurrent KET administration at different doses affects neuron damage and apoptosis; its effects in longer exposures in the juvenile period are also unclear. The present prospective randomized experimental study aimed to determine whether repeated (three times per day) KET administration at subanesthetic and anesthetic doses (5, 20, and 50 mg/kg) [33,34] for three consecutive days leads to undesired histopathological changes in the cerebral cortex, striatum, and hippocampus in juvenile rats ten days after the administration.

## 2. Materials and methods

This prospective, randomized experimental trial protocols were approved by the Ankara University Animal Experiments Local Ethics Committee (2020-36, 2020-7-6232). Rats were procured from Ankara University, School of Medicine, Experimental Animals and Research Laboratory. After the approval of the ethics committee, 32 Wistar Albino juvenile (30-day-old) male rats were randomized into four groups: 1 mg/kg serum saline (Group C), 5 mg/kg KET (Group 5), 20 mg/kg KET (Group 20), and 50 mg/kg KET (Group 50). Intraperitoneal (i.p.) KET [(Ketalar), ketamine HCl, 500 mg/10 mL injectable, Pfizer FE Pharmaceuticals, İstanbul, Turkey] or serum saline [0.9% sodium chloride (NaCl) solution] was administered to the rats with 23G, 0.5 mL injectors three times a day in three-h intervals for three days based on the randomly selected groups. The mean body weight change in rats throughout the experiment was 3.28 ± 0.11 g, and there was no statistical difference between the groups. Ten days after the last KET dose, the rats were sacrificed using anesthesia by sodium-thiopental (50 mg/kg Pental İ.E. Ulagay Pharmaceuticals, Turkey). Brain tissues were removed. Rats’ brain tissues were prepared and evaluated histopathologically in Gazi University, Faculty of Medicine, Department of Histology. This study started in 2020 and finished in 2021.

### 2.1. Histological studies

The cerebral cortex and striatum were fixed with 4% paraformaldehyde, and tissue processing was conducted with an automated tissue processor (TP1020, Leica Biosystems, Germany). Paraffin-embedded tissues were sectioned in 4-μm thickness. After deparaffinization, sections were rehydrated with decreasing alcohol series. Hematoxylin and eosin (H&E) staining was performed [35,36].

### 2.2. Cleaved caspase-3 immunohistochemistry

Hippocampi fixation and tissue processing were conducted for cerebral cortex and striatum samples. Sections were deparaffinized and dehydrated. Antigen retrieval was conducted with a citrate buffer at 60 °C overnight. After blocking endogen peroxidase activity, primary antibody (ab2302, 1:50) incubation was done at room temperature for an h. Sections were incubated with secondary antibodies and 3-amino-9-ethylcarbazole as a chromogen. Hematoxylin was used for the ground stain.

Sections were evaluated with Leica DM4000 Light Microscopy Image Analysis Systems and Image J (NIH, USA) software by two blinded histologists. Neuron number per area (mm^2^) and cell size (micrometer) were evaluated in the cerebral cortex.

Statistical analysis was conducted with SPSS 21 software (IBM), a p-value <0.05 was considered statistically significant. The Kolmogorov–Smirnov test was used for the assessment of normality. Based on data distribution, either the Kruskal-Wallis or ANOVA test was used to analyze the difference between groups with Bonferroni post hoc analysis [35,36].

## 3. Results

### 3.1. Effect of ketamine on the morphology of the cerebral cortex

**Figure 1:**
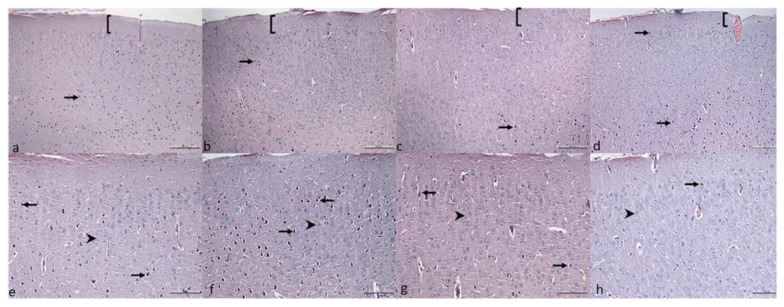
Hematoxylin & eosine-stained cerebral cortex micrographs (a–h). Group 5 mg/kg KET (a, e), Group 20 mg/kg KET (b, f), Group 50 mg/kg KET (c, g), Group C (1 mg/kg serum saline) (d, h). The scale bar represents 200 μm in the upper row and 100 μm in lower row. Square brackets ([) show molecular layer of the cortex, arrows show pyramidal neurons, arrowheads show granular cells.

Histological evaluation of cerebral cortex sections revealed nondistinct layers except the molecular layer in all groups. The pyramidal cells with dark basophilic cytoplasm had a multipolar shape. The granule cells with large, euchromatin nucleus and prominent nucleolus had a small amount of pale cytoplasm. Degenerative signs, such as eosinophilic neurons, contracted pale-staining nuclei, and gliosis, were not inspected in the sections. The cell size (μm) and the number of cells in the area (mm^2^) of the groups are demonstrated in Table 1. Figure 1 (a, b, c, d, e, f, g, h) shows the micrographs of the cerebral cortex.

### 3.2. Effect of ketamine on the morphology of the striatum

**Figure 2:**
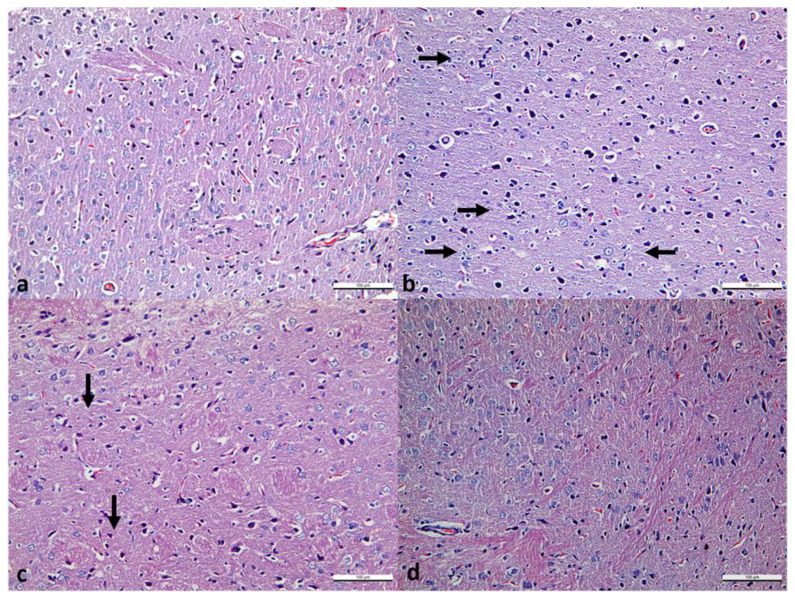
The Hematoxyline & eosine-stained striatum micrographs. Group 5 mg/kg KET (a), Group 20 mg/kg KET (b), Group 50 mg/kg KET (c), Group C (1 mg/kg serum saline) (d). The scale bar represents 100 μm. Arrows show gliosis.

Neuron bodies and neuropils were evaluated in striatum sections for all groups. The pyramidal and granule cells did not show degenerative changes. Mild gliosis was seen in Group 20, but gliosis was not prominent in Group 50. Figure 2 (a, b, c, d) shows micrographs of the striatum.

### 3.3. Immunohistochemistry results of ketamine effect on the CA1 region of the hippocampus

**Figure 3:**
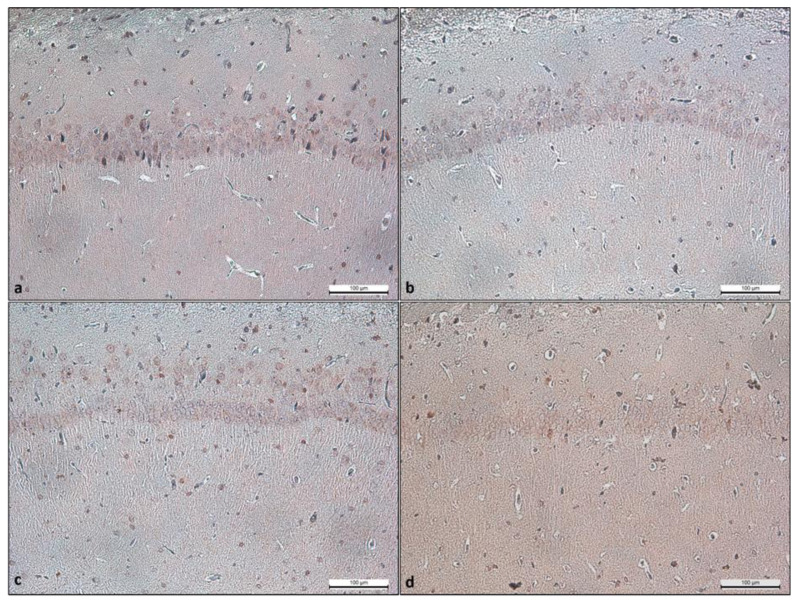
The cleaved caspase 3 immunohistochemical reaction micrographs. Group 5 mg/kg KET (a), Group 20 mg/kg KET (b), Group 50 mg/kg KET (c), Group C (1 mg/kg serum saline) (d). The scale bar represents 100 μm.

Cleaved caspase-3 was evaluated for apoptosis evaluation in the CA1 region of the hippocampus. Immune reactive cells were calculated per area (mm^2^). The mean and SD values of groups are presented in Table 2. Caspase-3 positivity increased with the KET dose and was lower in Group 5 than in Group C. However, the difference is statistically insignificant. Micrographs are presented in Figure 3 (a, b, c, d).

## 4. Discussion

The current experimental study findings revealed no differences between the groups in cell count or size in cortical morphometry. No degenerative changes were observed in pyramidal and granular cells in the striatum. Mild gliosis was observed in Group 20 and Group 50. Immunohistochemical analysis conducted to determine apoptosis in the CA1 region of the hippocampus revealed that caspase-3 positivity increased with the KET dose, although the difference was not statistically significant between the groups. While it was lower in Group 5 compared to Group C, it was similar in Group 20 and Group C and higher in Group 50 (p > 0.05).

Although several reports in the last three decades indicated that general anesthetic agents could lead to undesirable effects on the developing brain, others reported neuroprotective properties of KET [32].

Several studies conducted with various species, including nematodes and nonhuman primates, reported that early exposure to general anesthetic agents, especially KET, leads to neurodegeneration, which in turn leads to a decline in cognitive functions and socio-emotional development [14–23]. However, in most studies, KET was administered in the early stages of development, or the early postexposure findings were analyzed [32]. This raises a question about the nonacute effects of KET exposure in the juvenile period. This is because recurrent KET anesthesia in the clinic or chronic KET use due to other clinical indications could occur in early childhood and juvenile rather than fetal or neonatal periods.

Synaptogenesis in rats continues between 7–30 days postnatal [37,38]. One human year almost equals two rat weeks (365÷26.7 = 13.8 rat days) [39]. In this experiment to evaluate rapid brain development phase (juvenile phase), 30-day-old rats were preferred.

Cellular damage in neural tissue is determined by histopathological findings such as a decrease in cell count, change in cell size, eosinophilic neuron, pale-staining nucleus, gliosis, and increase in apoptosis. Damage to cerebral neurons is associated with regression in cognitive functions [40–42]. In vivo studies also reported that repeated KET administration leads to neuronal pyroptosis, apoptosis, and inflammation in the hippocampal region [43]. Experimental studies evaluating the neurotoxic effects of repeated KET administration on developing brain tissue have been performed on different animal groups and at different stages of neurodevelopment. Neurotoxic effects were evaluated by many histopathological, functional, or behavioral methods [14–23]. In the literature, it is reported that the KET dose, dose repetition, and KET exposure stage applied in animal studies are effective factors in neurological damage (Table 3). The time between KET exposure and histopathological analysis is also an important factor in determining the duration of cellular damage.

Zou X et al. analyzed the striatum, hippocampus, thalamus, and amygdala six h after KET administration in monkeys (20 mg/kg × 6 injections). They reported that KET led to an increase in caspase-3 positive apoptotic cell count; it was three-fold in the stratum, 2.5-fold in the hippocampus, 2.3-fold in the thalamus, and three-fold in the amygdala when compared to the control. The most obvious increase (10-fold) was identified in the frontal cortex [16]. Lyu et al. demonstrated that the administration of six 20 mg/kg KET doses at two-h intervals in postnatal seven-day-old Sprague-Dawley rats increased apoptosis in the hippocampus, and the apoptosis rate increased three h after administration and persisted even after six h [43].

In the current study, histopathological analysis of the rat brain tissues ten days after KET administration revealed no obvious cellular damage in the cortex. However, mild gliosis and an increase in caspase-3 in the CA1 region of the hippocampus were determined based on the KET dose, although the difference was not statistically significant in Group 20 and Group 50.

H&E staining was employed for morphological imaging and caspase-3 staining for immunohistochemical analysis to determine apoptosis in neural tissue [44,45].

The production of caspases that provide critical bonds for cellular regulation networks that control inflammation and necrosis as inactive zymogens and their acquisition of catalytic activities are under strict control. Activation of inflammatory caspases leads to an immune response through the release of active proinflammatory cytokines. Dysregulation of caspases underlies several diseases, including cancer and inflammatory disorders [46].

Soriano et al. reported that i.p. administration of KET (5, 10, and 20 mg/kg/dose) to postnatal seven-day-old rats five times (at 90-min intervals over six h) led to apoptotic necrosis in the cortical and thalamic regions of the baby rat brains [18]. Also, Zou et al. demonstrated that KET administration to seven-day-old rats led to an increase in neuronal apoptosis based on the dose and exposure period [47]. Similar findings were also reported in the experiment conducted with monkeys [16]. However, potential neurotoxic effects in these studies were analyzed six h after the final drug administration.

In our study, no degeneration was determined in cortex cells, while mild gliosis was identified in the striatum in Group 20 and Group 50. There was no statistically significant difference between immunoreactive cells in the CA1 region (p > 0.05). They were lower in Group 5 when compared to Group C, similar to Group C in Group 20, and higher in Group 50 when compared to Group C (Table 2).

Neurons in the CA1 region of the hippocampus are essential for spatial learning and memory functions. CA1-region neurons receive and process data from the entorhinal cortex or the CA3 region. The CA3 region is connected to the CA1 region via Schaffer collateral fibers. A solid CA3 and CA1-CA3 connection is required for reference memory. The cell count in CA1 and CA3 gradually increases after adolescence with spatial learning and memory functions [48]. Our experiment analyzed rat brain tissue, the cerebral cortex, and the striatum. The cell count in the cerebral cortex was measured. The pyramidal cells with dark basophilic cytoplasm had multipolar shapes. The granule cells with large, euchromatin nucleus and prominent nucleolus exhibited a small pale cytoplasm count. Degenerative signs, such as eosinophilic neurons, contracted pale-staining nuclei, and gliosis, were not determined in the sections. Neuron bodies and neutrophils were observed in striatum sections. The pyramidal and granule cells did not exhibit degenerative changes. Mild gliosis was observed in Group 20; however, it was not prominent in Group 50.

The main limitations of this experimental study were not measuring the blood KET level in the rats and not using methods other than histopathological methods to show cell damage to evaluate neurotoxicity.

## 5. Conclusion

Ten days after repeated KET administration in juvenile rats, 20 and 50 mg/kg KET doses had negative effects on the cerebral cortex, striatum, and hippocampus, although these changes were not obvious. Although there was no statistically significant difference between apoptosis in the hippocampus with KET administration compared to Group C, apoptosis decreased after the 5 mg/kg administration, was similar with the 20 mg/kg dose, and increased with the 50 mg/kg dose.

According to current data, it is not possible to avoid general anesthesia in clinical practice. In our study, repeated KET exposure did not significantly affect cerebral morphology and apoptosis in juvenile rat hippocampal cells, but KET doses are worth considering. The experimental design of this study means these results could not be totally adapted to human beings.

## Figures and Tables

**Table 1:** Cortical morphometric data.

Groups	Total cell per area (mm^2^) Mean ± SD	p values	Cell size (μm) Mean ± SD	p values
Group 5 mg/kg KET	692.06 ± 178.45	Group 20 mg/kg 1.000, Group 50 mg/kg 1.000, Group C 1.000	8.61 ± 1.02	Group 20 mg/kg 1.000, Group 50 mg/kg 0.239, Group C 1.000
Group 20 mg/kg KET	687.71 ± 132.47	Group 50 mg/kg 1.000, Group C 1.000	9.33 ± 0.97	Group 50 mg/kg 1.000, Group C 1.000
Group 50 mg/kg KET	731.12 ± 153.43	Group C 1.000	9.95 ± 1.62	Group C 0.667
Group C (1 mg/kg serum saline)	766.89 ± 214.13		8.93 ± 1.13	

Mean ± SD, the differences between groups were statistically insignificant p > 0.05

KET: Ketamine

**Table 2:** Caspase-3 positive cells per area (mm^2^).

Groups	Caspase-3 positive cells per area (mm^2^) Mean ± SD	p values
Group 5 mg/kg KET	130.65 ± 62.84	Group 20 mg/kg 1.000, Group 50 mg/kg 1.000, Group C 1.000
Group 20 mg/kg KET	143.89 ± 67.45	Group 50 mg/kg 1.000, Group C 1.000
Group 50 mg/kg KET	166.59 ± 30.87	Group C 1.000
Group C (1 mg/kg serum saline)	143.25 ± 73.12	

Mean±SD, the differences between groups were statistically insignificant p = 0.731

**Table 3:** Experimental steps and results of some experimental ketamine studies on animals.

Animal and stage	Method and doses of ketamine	Neuronal toxicity or injury	Evaluation method	Outcome
Postnatal 7-day old rats	Subcutan 7 doses one every 90 min, of either saline, 10 mg/kg or 20 mg/kg ketamine, or a single dose of 20 mg/kg ketamine	Neurotoxicity Neuronal apoptosis	Silver staining histochemical results Fluoro-Jade B (FJ-B)	The repeated doses of 20 mg/kg ketamine increased the degenerating neurons in the dorsolateral thalamus [14]
Postnatal 7-day-old Sprague-Dawley rats	Intraperitoneal injections of a single dose of ketamine 25, 50, and 75 mg/kg, repeated doses of ketamine 25 mg/kg at 90-min intervals over 9 h	Degeneration of neurons	10 brain regions using the stereological disector method Cupric-silver stain	Repeated doses of ketamine (25 mg.kg^−1^) at 90-min intervals over 9 h increased degenerating neurones in seven out of 10 brain regions [15]
Postnatal day 5 or 6 newborn rhesus monkeys	Intravenously for 3, 9, or 24 h to maintain a steady anesthetic plane, followed by a 6-h withdrawal period	Neuronal cell death in layers II and III of the frontal cortex Caspase-3- and Fluoro-Jade C-positive neuronal profiles in hippocampus, thalamus, striatum, and amygdala areas	Silver and Fluoro-Jade C stains and caspase-3 immunostain	No significant neurotoxic effects occurred if the anesthesia duration was 3 h but anesthetic durations of 9 h or greater are associated with significant brain cell death in the frontal cortex [16]
Rhesus monkeys (N = 3 for each treatment and control group) at three stages of development 122 days of gestation and 5 and 35 postnatal days	Intravenously for 24 h to maintain a surgical anesthetic plane, followed by a 6-h withdrawal period	Apoptotic and necrotic cells in the frontal cortex Electron microscopy for nuclear condensation and fragmentation in some neuronal cells, and cell body swelling	Caspase 3-Fluoro-Jade C- and silver stain	A shorter duration of ketamine anesthesia (3 h) did not result in neuronal cell death in the 5-day-old monkey. Earlier developmental stages appear more sensitive to ketamine-induced neuronal cell death than later in development [17]
Postnatal day 7 rat pups	Intraperitoneal 5 doses of either saline or ketamine (5, 10, and 20 mg/kg/dose) at 90-min intervals over 6 h. Primary neurons were exposed to varying concentrations of ketamine to determine the dose and duration effects	Cyclin D1, cyclin-dependent kinase 4, and E2F1, Bcl2-interacting mediator of cell death, and activated caspase-3	The expression of cell cycle proteins, mediator of cell death, and activated caspase-3 was determined	Ketamine induces aberrant cell cycle reentry, leading to apoptotic cell death in the developing rat brain [18]
Fetal Sprague Dawley rats	The primary hippocampal neurons isolated form fetal Sprague Dawley rats were treated with 0, 50, 100, and 500 μM ketamine for 4 days	mTOR signaling pathway and apoptosis of rat hippocampal neurons (TUNEL)	The expressions of mTOR signaling pathway and apoptosis-related proteins in hippocampal neurons were examined by qRT-PCR and Western blot	Ketamine could dose-dependently promote the apoptosis of rat hippocampal neurons with upregulation of p-mTOR and its downstream regulators [19]
Postnatal day 7 Sprague-Dawley rat pups	Intraperitoneal ketamine treatment 25, 50, and 75 mg/kg, for three consecutive days	Hippocampal neurodegeneration and behavioral deficits in adulthood	Protein kinase C-Gamma (PKCγ), extracellular signal regulated kinase (ERK)1/2 and Bcl-2 expression	Ketamine at a dose of 75mg/kg in the developing brain results in hippocampal neurodegeneration and persistent learning and memory impairment, which is associated with the PKCγ-ERK signaling pathway [20]
Pregnant rats (P19)	Intravenous injection of ketamine (200 mg/kg) for 3 h	Fetal hippocampus tissue Fetal brain tissue Cultured PC12 cells in vitro to determine the relationship between ROS, autophagy, and apoptosis	Total Antioxidant Capacity, Reactive Oxygen Species, and Malondialdehyde. Changes in the levels of Cleaved-Caspase-3, Beclin-1, B-cell lymphoma-2, Bcl-2 Associated X Protein, Autophagy-related gene 4, Atg5, p62, and marker of autophagy Light Chain 3.	Anesthesia with ketamine in pregnant rats may increase the rate of autophagy and apoptosis in the fetal hippocampus and the mechanism may be through inhibition of antioxidant activity and ROS accumulation [21]
Postnatal day 10 male mice	Subcutan a single dose of 7.5 g/kg ketamine One h after ketamine exposure, mice were whole body irradiated with 50–200 mGy gamma radiation. Behavioural observations were performed at 2, 4, and 5 months of age. At 6 months of age, cerebral cortex and hippocampus tissue were analysed for neuroprotein levels	Impaired learning and memory	Morris Water Maze	Coexposure to IR and ketamine can aggravate developmental neurotoxic effects at doses where the single agent exposure does not impact on the measured variables [22]
7 days old ICR mice	Subcutan 1.25, 2.5, 5, 10, 20, and 40 mg/kg ketamine in 0.9% NaCl or with 0.9% NaCl alone as control Righting reflex testing was performed and mouse brains were examined at 24, 48, and 72 h and 7 days after injection	DNA fragmentation and apoptosis	The number of degenerating neurons was measured using silver staining	The administration of ketamine in a clinically relevant single dose to 7-days old mice induced apoptosis in the sensorimotor cortex and cerebellum. This effect was dose-dependent and long lasting [23]
